# Report on Clinics Given at Ohio State Dental Society

**Published:** 1900-02-15

**Authors:** 


### REPORT ON CLINICS GIVEN AT OHIO STATE DENTAL SOCIETY.

### GOLD FILLING BY HAND PRESSURE.

BY DR. E. D. SCHEBLE, TOLEDO.

Dr. Scheble has used this method for sixteen years and uses all kinds of foils, preferably filling cavities with soft gold. The patient operated upon presented a mesial cavity in the superior left lateral incisor, quite extensive, reaching from cervical margin to near the incisal angle. The gold used was S. S. White's No. 4 soft gold. The instruments were ordinary serrated pluggers of simplest forms; large ebony handles. The pressure was heavy, but the patient claimed there was no pain. The filling when completed had the appearance of being a good one. He used fine-pointed instruments to finish around edges.

### FILLING WITH TIN AND GOLD.

BY DR. C. I. KEELY, HAMILTON.

The patient was a gentleman of thirty-five, who presented two cavities in the superior left bicuspids, a distal cavity in the first and mesial cavity in the second.

Dr. Keely, after preparing cavity first in the mesial portion of second bicuspid, applied the matrix, allowing it to be loose, so as to allow the tin to be flush or extend a little over edge of the cavity. He used Robinson's fibrous foil, building up the cavity about two-thirds with this material, his favorite instrument being a fine-pointed, smooth-tipped plugger, malleting with a lead mallet of considerable weight. Ney's soft gold was then used, annealing in an alcohol flame.

Dr. Keely is quite fortunate in being able to mallet with his left hand, thus having his right for the manipulation of the plugger.

His reasons for the use of tin are: 1st. The therapeutic value due to the chemical action which takes place between tin and tooth, also on surface of tin, thus lessening decay; 2d. The time saved in an operation, as cavity is more quickly filled with tin.

One interesting feature was his method of starting the gold. The last piece of tin was adapted so as to leave a little pit at the linguo-pulpal angle of the cavity, in which it was quite easy to start the gold. About two-thirds of the cavity was filled with tin (this varying, of course, according to the cavity), when he finished with gold.

The matrix was then removed and the tin burnished over the edges of cavity and at cervical margin, the filling then being finished with strips lubricated with soap, he used this in preference to oil, on account of disagreeable odor of the latter. Moore's disks were then used in finishing. The distal filling in the first bicuspid was then made in a similar manner.

### CONTOUR FILLING, USING WOODWARD MATRIX.

BY DR. J R. OWENS, CLEVELAND.

The case supplied Dr. Owens was not that which best demonstrated the Woodward matrix. It was a distal cavity of the left inferior second bicuspid; the first molar that should adjoin the bicuspid had been removed and a space equal to one-third of the molar remained. The case was a large cavity with the pulp exposed. This incidentally required immediate removal of pulp and root filling. The pulp was anesthetized with hydrochlorate of cocain, injected by pressure, and root and pulp cavity filled with oxychlorid of zinc. In adjusting the Woodward matrix, the space between it and the second molar was packed with gutta-percha, and when the gutta-percha was hard the screws of the matrix were brought to bear upon the gutta-percha; this, however, not being as firm and stable as a tooth would be, a ligature was placed about the bicuspid and upper part of the matrix to hold it firmly in place. The operation was entirely successful.

### GOLD AND TIN WORK.

BY DR. H. L. AMBLER, CLEVELAND.

The exhibition was of gold and tin work, using Dr. Ambler's tin foil. His exhibition consisted of: 1st. Filling in natural tooth; 2d. In steel matrices; 3d. Filling with pure tin base and gold face; 4th. Filling of tin and gold folded in alternate layers; 5th. Tin fillings made with an ivory plugger; 6th. Tin and gold fillings made with a tape of tin and gold folded in alternate layers, filling made with an ivory plugger; 7th. Gold fillings in natural teeth, made with an ivory-pointed plugger (Shumways).

Dr. Ambler, in his own practice, uses serrated pluggers.

### NEW INSTRUMENTS FOR REMOVING TISSUE COVERING PARTIALLY ERUPTED TEETH.

BY J. R. BELL, CLEVELAND.

The set consists of four instruments, for which Dr. Bell, we understand, has a patent, and he claims they are the first that have been made which will successfully remove that soft tissue overlying a partially erupted third molar. The parts are first anesthetized and the lance is drawn from within outward, cutting two transverse grooves or slits about as deep as the second molar. Then he takes up this flap with a pair of forceps and uses his curved probe-pointed scissors, cutting out the flap and exposing the tooth. The instruments are entirely new in design and are made by Fred. Hoslan, No. 83 Pulaski St., Brooklyn, N. Y.

### CONTINUOUS GUM WORK.

BY DR. W. T. JACKMAN, CLEVELAND.

This clinic was packing and baking body and enamel for a continuous gum denture. He used the electric furnace. There was nothing new in this clinic, the object of the clinician being only to stimulate the members of the dental profession to do more porcelain work, especially of this class. He claims we could do more of this kind of work if we were prepared to do it and would show the beauty and utility of it to our patients. To do this he claims that it is necessary to have an office piece to show, for it is utterly impossible to have the patient understand what you mean by continuous gum work unless he can actually see it.

### A NEW ALUMINUM SOLDER.

BY DR. J. FRANK COOK, TOLEDO.

This was a demonstration of the use of aluminum solder in plate work. The solder was of sandy consistency, containing the flux in its make-up; to use same it is necessary to use pressure to cause the two pieces of aluminum to adhere or properly solder. This is a great disadvantage in its use; the clinician himself had difficulty in making the pieces stick together.

### CROWN AND BRIDGE-WORK MADE WITH MASON DETACHABLE TEETH.

BY DR. W. O. HULICK, CINCINNATI.

The exhibition consisted of several specimens of crown and bridge-work, as made by the use of Mason's detachable teeth, which looked well and seemed to be very strong.

The system appears to be efficient and is probably well worth a trial at least.

### A METHOD OF MAKING GOLD FILLINGS IN ARTIFICIAL TEETH.

BY DR. S. M. WEAVER, CLEVELAND.

The idea in filling artificial teeth is to get rid of that "horrid false tooth look" of which the patients are very likely to complain. After the tooth is selected and ground to fit in the proper place, the tooth is placed in the mouth and the shape of the filling in the natural teeth are carefully noticed and the same is reproduced in the artificial tooth by grinding the proximal faces with a small, round-cornered corundum wheel; when this is of the proper shape the margins are polished with a fine stone or disk, the same as you would in a natural tooth. A gold backing of 24k, 32 gauge, broad enough to be burnished up into the cavity, which can be done with the shank of a small round instrument, is then adjusted. The surplus gold is then trimmed off, leaving enough extra to extend a trifle above the margins of the porcelain. A

platinum backing of 32 gauge is then placed over the gold, allowing it to extend out flush with the contour of the back of the tooth, leaving it slightly flush. The pins are then split to hold backing into place. There is now a V-shaped space to be filled in with 22k gold; to do this, place the tooth in a pair of self-holding soldering pliers with a small piece of asbestos over the porcelain to keep the oxide of iron from staining it. Wipe the cavity with a very little borax cream and hold above the Bunsen burner, porcelain side down, and then gradually carrying it into flame, when the facing becomes red-hot turn it over and place the small pieces of gold already prepared into the cavity, taking pains to warm them in the flame before touching the tooth as the chill might check the facing; care must be taken not to remove it from the flame until the soldering is complete. Next place the brush flame of the blow-pipe on and melt down the gold gently, being sure you have enough material on to restore the contour. To prevent the possibility of checking, throw it into warm, dry plaster and allow it to cool. When cool grind and fit the case and you will have the contour of the former tooth reproduced. This method can be used in crown, bridge and plate teeth.

### THE FELL METHOD OF FORCED RESPIRATION.

BY DR. HAMLIN BARNES, WELLSVILLE.

He showed the method of using forced respiration as designed and described by Dr. Fell, of Buffalo. Dr. Barnes exhibited apparatus for sustaining life in cases of asphyxiation or poisoning and in some cases of drowning; he also pointed to some cases in the experience of Dr. Fell where he had saved lives of a number of people where the ordinary means of artificial respiration entirely failed. Dr. Barnes claims that life can be sustained for an almost indefinite time by keeping the patient supplied with oxygen. In some cases he advises the reinforcement of the atmospheric air by the addition of pure oxygen, the apparatus being so constructed as to allow connection with cylinder of pure oxygen.

### THE USE OF COMPRESSED AIR FOR MEDICATING PURPOSES.

BY DR. IRA BROWN, CLEVELAND.

Dr. Ira Brown gave a demonstration of the use of compressed air attached to an atomizer with proper shaped nozzles to supply medicaments to the spaces around the teeth and to alveolar pockets that may contain pus. Dr. Brown claims that by this method even the extreme ends of the roots may be reached. He has a tank which he fills with an ordinary bicycle pump. He uses a weak solution of formaldehyd, although any good antiseptic can be used.

### IMPRESSIONS AND CASTS FOR USE IN ORTHODONTIA.

BY DR. V. E. BARNES, CLEVELAND.

The materials used were quick-setting impression plaster and the Detroit modeling compound. In taking impressions care is taken to get accurate impressions of the teeth and gums. They are three in number, superior, inferior and the bite, taken in composition; there is also a face model taken of one side of face, in plaster. The face is protected with a coating of vaseline, ear is protected with cotton, and the eye protected with rubber and the cloth vaselined. The face impression is waxed and varnished ready to fill.

Casts are then made of the face impression, also the superior, inferior and bite impressions. They are symmetrically trimmed and treated with stearin, to give a polish and wearing qualities.

### REMOVAL OF TOOTH-PULP BY MEANS OF PRESSURE ANESTHESIA.

BY DR. OTTO ARNOLD, COLUMBUS.

Dr. Arnold presented an interesting clinic in anesthetizing the pulp of a superior central incisor. He applies a saturated solution of hydrochlorate of cocain in alcohol on cotton in the cavity which has an exposure; over this he places soft vulcanite, and by using pressure is enabled to force the cocain into the pulp, anesthetizing in this case in about ten minutes. In the case presented Dr. Arnold found a constriction in the root, about a line from the apex, and it caused him to make a second application. He was enabled to clear the root canal to the apex with very little pain to the patient.

### USE OF THE AUTOMATIC PLUGGER.

BY DR. W. T. M'CLEAN, CINCINNATI.

Dr. W. T. McClean, Cincinnati, demonstrated the use of the automatic plugger. He filled a cavity in a superior lateral incisor, distal-proximal surface. The cavity was accessible, but quite extensive. Dr. McLean works rapidly and uses the automatic plugger altogether.

### GOLD-LINED CAVITY AND TIN FILLING.

BY DR. C. R. BUTLER, CLEVELAND.

This demonstration consisted of a gold-lined anterior wall of cavity, in the lateral surface of a superior central incisor, body of filling being made of tin—a considerable portion of palatal wall was broken away, which was restored in contour with tin. The principal object sought was to show a *saving* filling in case of frail and badly-decayed teeth. (Not a cheap operation.) The six or eight anterior teeth are amenable to this kind of treatment, with no unsightly discoloration of tooth structure by the oxidation of the tin.

### BLEACHING BY PYROZONE AND ELECTRIC CURRENT.

BY DAVID STERN, CINCINNATI.

A badly-discolored central incisor was bleached by the use of the electric current and a solution of pyrozone. He opened into the tooth, the root of which had been filled; he did not remove all of root filling, but cleaned the tooth thoroughly, applied the pyrozone on some cotton and then applied the current. It was a successful clinic.

### AN INGENIOUS METHOD OF TAKING IMPRESSIONS.

BY DR. J. B. SNYDER, BRYAN.

Take first an impression with modeling compound, then examine the mouth for hard places; cut out impression at these points, inserting soft putty. Then resoften the compound and take another impression. In this way you get a better impression of all portions of the mouth.

### ROLLED GOLD WIRE FOR POOR JOINTS.

BY DR. S. D. RUGGLES, PORTSMOUTH.

Dr. Ruggles presented a good thing in gold wire rolled out to an extreme thinness; this he uses in those crowns where a poor joint is made between facings and band. He burnishes this wire into space thus formed and then solder is easily flowed over same.

### A WIRE-DRAWING MACHINE.

BY DR. J. F. STEPHAN, CLEVELAND.

This machine consisted of a bicycle chain which was gripped by a device and was operated by crank arrangement, thus getting a steady pull. It was also arranged so as to release grip to take new hold of wire. The whole was mounted on a wooden horse.

### WIRE-DRAWER—GOLD-POLISHER.

BY DR. W. S. LOCKE, CINCINNATI.

Dr. Locke presented a machine for drawing wire, which, while very inexpensive, is very powerful, being constructed on the plan of a walking board. He also presented a method, which he claimed was original with Dr. C. P. Sweeney, of Cincinnati, of burnishing gold-plating with a brass brush wheel and licorice water, which does the work very quickly and perfectly.

### MAKING-OVER OLD INSTRUMENTS.

BY DR. E. B. LODGE, CLEVELAND.

Dr. Lodge demonstrated how he makes use of his old instruments by making them over. He claims he makes better excavators than he can buy, as he makes them to suit each case. His was a very interesting demonstration, as well as being something which we can profit by. We all have spare time occasionally, when it could be used profitably in this way.

### REFINING GOLD.

BY DR. A. A. KUMLER, CINCINNATI.

Dr. A. A. Kumler refined gold in the old manner, dissolving scraps in nitro-hydrochloric acid and precipitating same as pure gold. His method while old was very interesting.

